# Measurement of in-plane elasticity of live cell layers using a pressure sensor embedded microfluidic device

**DOI:** 10.1038/srep36425

**Published:** 2016-11-04

**Authors:** Chien-Han Lin, Chien-Kai Wang, Yu-An Chen, Chien-Chung Peng, Wei-Hao Liao, Yi-Chung Tung

**Affiliations:** 1 Academia Sinica, Research Center for Applied Sciences, Taipei, 11529, Taiwan; 2 Tamkang University, Department of Civil Engineering, New Taipei City, 25137, Taiwan; 3 National Taiwan University, Department of Mechanical Engineering, Taipei, 10617, Taiwan

## Abstract

In various physiological activities, cells experience stresses along their in-plane direction when facing substrate deformation. Capability of continuous monitoring elasticity of live cell layers during a period is highly desired to investigate cell property variation during various transformations under normal or disease states. This paper reports time-lapsed measurement of live cell layer in-plane elasticity using a pressure sensor embedded microfluidic device. The sensor converts pressure-induced deformation of a flexible membrane to electrical signals. When cells are cultured on top of the membrane, flexural rigidity of the composite membrane increases and further changes the output electrical signals. In the experiments, human embryonic lung fibroblast (MRC-5) cells are cultured and analyzed to estimate the in-plane elasticity. In addition, the cells are treated with a growth factor to simulate lung fibrosis to study the effects of cell transformation on the elasticity variation. For comparison, elasticity measurement on the cells by atomic force microscopy (AFM) is also performed. The experimental results confirm highly anisotropic configuration and material properties of cells. Furthermore, the in-plane elasticity can be monitored during the cell transformation after the growth factor stimulation. Consequently, the developed microfluidic device provides a powerful tool to study physical properties of cells for fundamental biophysics and biomedical researches.

Cellular microenvironment plays a critical role in regulating biological activities under various normal and pathological conditions. To understand interactions between cells and their microenvironments, investigation on physical properties of cells becomes essential. Functions of cells are determined by their structures, and the structural organization of cells can be characterized by various physical properties. Elasticity is one of the most important physical properties, but not well studied due to technical limitations. For instance, several studies have shown a reduction in cell elasticity with increasing metastatic efficiency in human cancer cell lines^1^,^2^,^3^,^4^. Therefore, by investigating physical properties of cells, malignant and non-malignant cells can be reliably distinguished while normal and cancerous cell morphologies are similar^5^. In addition, different subpopulation cells can be rapidly sorted by their elasticity^6^. Consequently, measurement of cell elasticity is an essential task in biomedical research.

A number of studies have attempted to characterize elastic properties such as Young’s modulus or shear modulus through whole-cell or spatially confined (point on a cell) approaches. For example, whole-cell elasticity in suspension can be measured by micropipette aspiration^7^,^8^,^9^. In another research, shear modulus of human erythrocyte membrane can be estimated using optical tweezers^10^. Optical stretcher is exploited to measure elasticity of biological cells without mechanical contact^11^. Also, physical characteristics such as elastic behaviour, viscous response and contractile behaviours of adherent fibroblast cells can be measured using microplate manipulation and elastic substrate methods^12^,^13^. In addition, viscoelastic response and related physical properties of cells can be observed by monitoring fluorescent nano-beads injected into fibroblast cells^14^.

Among various characterization methods, atomic force microscopy (AFM) has been broadly utilized. It provides a direct access for researchers to obtain nano-scale topographical and physical information about cells. AFM has several advantages such as high spatial resolution, and can be operated in aqueous solution that allows live cells be analyzed in their physiological environments. The elasticity (Young’s modulus) of various cells such as endothelial cells, leukocytes and fibroblasts has been characterized using AFM^15^,^16^,^17^,^18^,^19^,^20^,^21^. Due to the working principle of probe indentation, most of the Young’s modulus are measured in the direction normal to cell membranes or substrate planes. However, material properties and interactions between cells or cellular motions are considered to be anisotropic because of their anisotropic configuration^22^,^23^. In various physiological activities, such as: lung expansion during inhalation and vasodilation, cells experience stresses along their in-plane direction when facing substrate deformation. Numerous studies have been conducted to investigate cell behavior under various substrate deformation^24^,^25^,^26^,^27^. Therefore, elasticity along in-plane direction also plays important roles in regulating biological activities. In addition, cells can be easily damaged, and physical properties of cells may be altered during AFM measurements. As a result, development of a convenient system for investigating live cell properties along in-plane direction within a certain period (in order of days) is highly desired.

In order to overcome the limitations of the existing characterization instruments for cell elasticity measurement, we develop a novel microfluidic device to study the in-plane elasticity of cell layers in this paper. Microfluidic devices have been shown to provide controlled microenvironments for *in vitro* cell studies and analysis^28^,^29^. It has advantages of small sample and reagent volumes, low power requirements and low fabrication costs. In this paper, the microfluidic device is made of polydimethylsiloxane (PDMS), a silicon-based elastomeric material with several advantages, including: cost-effective fabrication, great manufacturability, mechanical robustness, and disposability. Additionally, PDMS is non-toxic to cells, gas permeable and has excellent optical properties including optical transparency^30^,^31^,^32^,^33^. The microfluidic cell culture device is designed with an embedded pressure sensor for measuring the Young’s modulus along the substrate on which cells are attached. The pressure sensor is constructed using electrofluidic circuit that can be seamlessly integrated into the microfluidic device without sophisticated fabrication process and complex instrumentation^34^,^35^,^36^. The in-plane elasticity of cell layers can be estimated from the electrical signal output of the pressure sensor, which makes automated measurement feasible. Furthermore, the pressure sensor provides excellent long-term and temperature stability, which are desired for cell culture applications.

In experiments, the developed microfluidic device is fabricated using soft lithography, and the pressure sensor is calibrated before cell experiments. In the cell experiments, human embryonic lung fibroblast (MRC-5) cells are cultured and analysed to estimate their in-plane elasticity using the device for demonstration. Furthermore, the MRC-5 cells are treated with a growth factor to simulate lung fibrosis to study the effects of cell transformation on elasticity variation. In addition, AFM measurement is also performed for comparison in this paper. The developed microfluidic device offers an excellent tool to study in-plane elasticity of cells within a certain period with simple instrumentation and easy operation. The device provides a promising technique to study cell physical properties and cell-cell interactions under various physiological conditions, and greatly helps biologists investigate essential biophysical questions.

## Materials and Methods

### Microfluidic device design and fabrication

**Figure 1:**
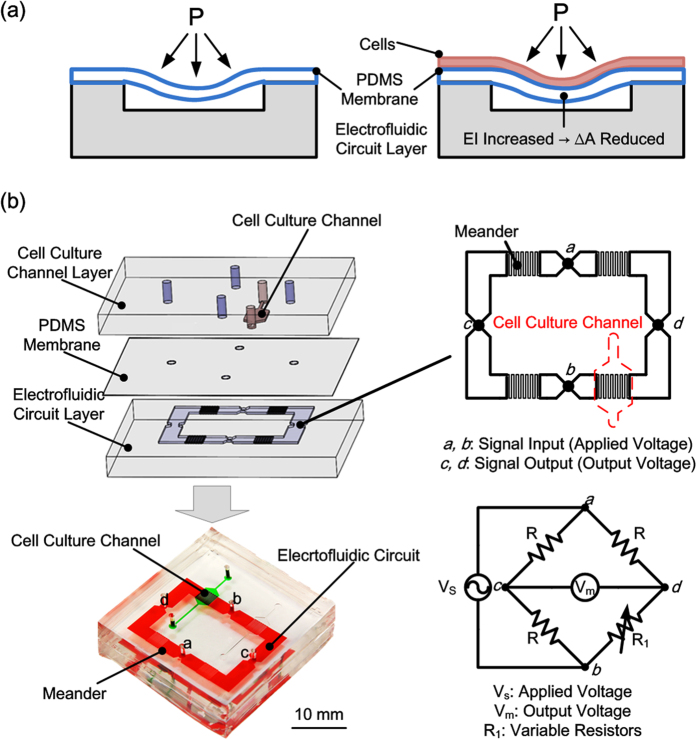
(**a**) Operation principle of the microfluidic device capable of measuring in-plane elasticity of cell layers. (**b**) Schematic and an equivalent electrical circuit of the microfluidic device with an embedded electrofluidic pressure sensor, and a photo of the fabricated device filled with colored food dyes.

In order to measure the in-plane elasticity of cell layers, a microfluidic device with an embedded pressure sensor based on electrofluidic circuits is developed in this paper. In brief, an electrofluidic circuit is an electrical circuit constructed using ionic liquid (IL)-filled microfluidic channels. IL is salt in liquid state, and has various unique material properties, including: electrical conductivity, low vapour pressure, and thermal stability. By designing the channel geometries, various electrical components, including: resistors, variable resistors, and switches, can be constructed^35^. The electrofluidic pressure sensor is constructed based on the electrofluidic variable resistors and a Wheatstone bridge circuit. The variable resistor is controlled by pressure-induced deformation of an elastic membrane. When the membrane is pressurized, its deformation alters cross-sectional area of an IL-filled microfluidic channel as shown in Fig. 1(a). Electrical resistance value in a circuit is changed due to the variation of its cross-sectional area. As a result, the magnitude of the applied pressure can be estimated by measuring electrical property change of the circuit. In order to measure the in-plane elasticity of cells, an electrofluidic pressure sensor is characterized with and without cells cultured on top of the membrane. When cells are cultured on top of the membrane, the resulted flexural rigidity of the composite membrane is increased and further affects the membrane deformation as shown in Fig. 1(a). Therefore, difference of the flexural rigidity between the membrane with and without cells cultured on it can be estimated by characterizing the electrical resistance of the variable resistor. In order to accurately measure the resistance change, a Wheatstone bridge circuit is exploited to construct the pressure sensor. The Wheatstone bridge architecture provides the pressure sensor several desired advantages for cell culture applications, including: great long-term stability (more than a week), temperature stability (up to 100 °C), and sensor linearity^34^.

In this paper, the electrofluidic pressure sensor embedded microfluidic device consists three PDMS layers: a cell culture channel layer, a PDMS membrane and an electrofluidic circuit layer as shown in Fig. 1(b). On the top microfluidic channel layer, a cell culture channel with a single inlet and a single outlet is designed to culture cells for the in-plane elasticity measurement. On the bottom electrofluidic circuit layer, four electrofluidic resistors, including the pressure sensing one, designed with identical geometries are arranged as a Wheatstone bridge circuit as shown in Fig. 1(b). In the experiments, an electrical voltage signal, *V*_*S*_, is applied from the signal inputs *a* and *b*, and the voltage across the bridge circuit, *V*_*m*_, is measured from the outputs *c* and *d*.

The pressure-sensing resistor is aligned to the cell culture channel on the top layer. The entire device is fabricated using the well-developed soft lithography replica molding technique^33^,^37^. The top cell culture channel and bottom electrofluidic circuit layers are made of commercially available elastomeric material, PDMS (Sylgard 184, Dow Corning, Midland, MI), cast against silicon wafer molds having SU-8 microfluidic channel features with thickness of approximate 180 μm patterned by conventional photolithography. The fabricated molds are then silanized by 1H,1H, 2H,2H-perfluorooctyl trichlorosilane (97%) (L16606, Alfa Aesar, Ward Hill, MA) in a desiccator for more than 30 minutes to prevent undesired bonding of PDMS to the mold. PDMS prepolymer (Sylgard 184, Dow Corning, Midland, MI) with 1:10 (v/v) curing agent to base ratio is poured on the molds and cured at 60 °C for more than 4 hours. The PDMS membrane, with a thickness of 80 μm, is prepared by spinning the PDMS precursor onto a silanized silicon wafer. Medium inlet and outlet of the cell culture channel, signal input and output holes for electrofluidic circuits on the top cell culture layer are punched using a biopsy punch with a diameter of 2 mm. Then three layers are permanently bonded together using oxygen plasma surface treatment. The entire assembled device is placed on top a hot plate with temperature of 120 °C overnight to promote the bonding between PDMS layers and cell compatibility. Finally, the bottom electrofluidic circuit layer is filled with ionic liquid, 1-ethyl-3-methylimidazolium dicyanamide (H26901, Alfa Aesar).

### Mechanical model and theoretical derivation

In order to estimate the in-plane elasticity of cell layers from the pressure sensor output voltage variation, a theoretical model based on solid mechanics and basic circuit theories are derived in this paper. The electrical resistance of each resistor in the electrofluidic circuit layer can be written as:


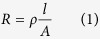


where *ρ* is the resistivity, *l* is the length of the conducting channel, and *A* is the cross-sectional area of the electrofluidic channel. When the applied pressure deforms the membrane above the electrofluidic circuit layer, the cross-sectional area of conducting channel is reduced and the resistance of the resistor is changed. As a result, the change of the resistance, *ΔR*, can be derived as:


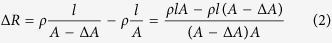


Since the change of cross-sectional area *ΔA* is much less than the original area *A*, the change of the resistance can be simplified as:


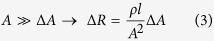


**Figure 2:**
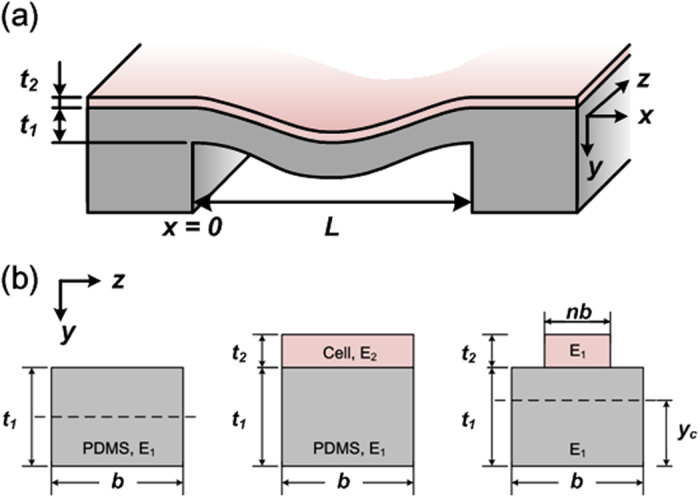
(**a**) The simplified beam with fixed end boundary conditions model to simulate the PDMS membrane deformation. (**b**) Transformed section for composite beam composed of PDMS and a layer of cells to estimate resulted moment of inertia.

In order to estimate the pressure-induced area change, *ΔA*, the PDMS membrane can be modeled as a fixed-fixed beam with fixed end boundary conditions as shown in Fig. 2(a). From the beam theory, the governing equation and the boundary conditions are written as:


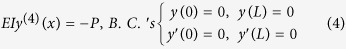


where *E* is the Young’s modulus of the beam material, *I* is the moment of inertia, *P* is the pressure applied on the membrane and *L* is the width of the electrofluidic circuit channel designed to be 150 μm in the experiments. The displacement equation of membrane in the vertical direction, *y*, can be derived as:


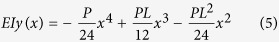


By integrating the displacement equation of membrane, the area variation, *ΔA*, can be given as:


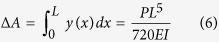


Consequently, the resistance change, *ΔR*, is:


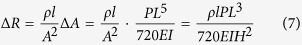


where *H* is the height of the electrofluidic circuit channel designed to be 70 μm.

In the electrofluidic circuit layer, four electrofluidic resistors are arranged as a Wheatstone bridge circuit. According to Kirchhoff’s laws, the measured voltage, *V*_*m*_, can be derived as:


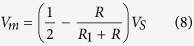


Since the four resistors have identical geometries (i.e. *R*_*1*_ = *R*); therefore, the measured output voltage is ideally zero before the pressurization. After applying the pressure in the channel facing to the variable resistor, the resistance of the resistor is changed due to the PDMS membrane deformation:


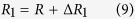


Combining equations (8) and (9), the measured voltage across two arms of the Wheatstone bridge can be written as:


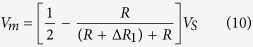


For small deformation of the membrane, *∆R* is much less than *R*, and *V*_*m*_ can be approximated as:


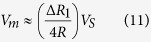


According to the solid mechanics derivation in equation (7), the relationship between the applied pressure and the measured voltage can be given by:


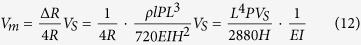


The equation shows that the measured voltage is inversely proportional to the membrane elasticity and its inertia. With the same applied pressure, deformation of the membrane decreases as a result of the larger flexural rigidity (*EI*), and the measured voltage becomes lower. Therefore, once cells adhere to the PDMS membrane, the flexural rigidity of the membrane is increased, and the corresponding deformation of the membrane under the same applied pressure is smaller comparing to the membrane without cell adhesion.

According to the beam theory, the cell-adhered membrane can be modeled as a composite material, and the ratio of Young’s modulus of two materials *n* is:


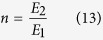


where *E*_*2*_ and *E*_*1*_ are the Young’s modulus of cells and PDMS, respectively. By multiplying the width of the cell layer *b* with *n*, two dissimilar materials, cells and PDMS, can be transformed as the same material with different widths, as shown in Fig. 2(b). In addition, the inertia of the original beam is changed due to the additional cell layer. Therefore, the moment of inertia of the transformed beam, *I*_*c*_, becomes:


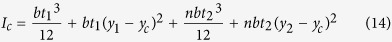


where *t*_*1*_ and *t*_*2*_ are the thickness of PDMS membrane and cell layer, respectively; *y*_*1*_ and *y*_*2*_ are the positions of each mass center calculated from the bottom of the beam, respectively. The mass center of the transformed beam *y*_*c*_ can be written as:


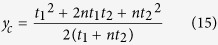


In order to precisely measure the in-plane elasticity of cell layers, the pressure sensor sensitivity, instead of output voltage under specific pressure, is measured to minimize the setup variation in each measurement. In the experiments, the sensitivity is calculated as ratio of the output voltage variation to a unit of applied pressure change. In the experiments, the output voltages under various applied pressures are measured, and the linear regression is applied to calculate the sensitivity. Derived from equation (12), the sensitivity can be written as:


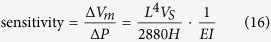


Comparing the pressure sensitivities of the same device with and without cells cultured on the membrane, the ratio of the elasticity between the membrane and the composite membrane, *n*, can be obtained using the equation:


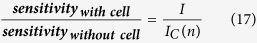


The ratio of pressure sensor sensitivities between the same device with and without cells adhered to the membrane in the culture channel equals to the ratio of the moments of inertia between the original and transformed cross-sections. Through the presence of the measured moment of inertial of the transformed section, which is a function of *n*, the in-plane Young’s modulus of cell layers can be determined experimentally.

### Pressure measurement and data acquisition

**Figure 3:**
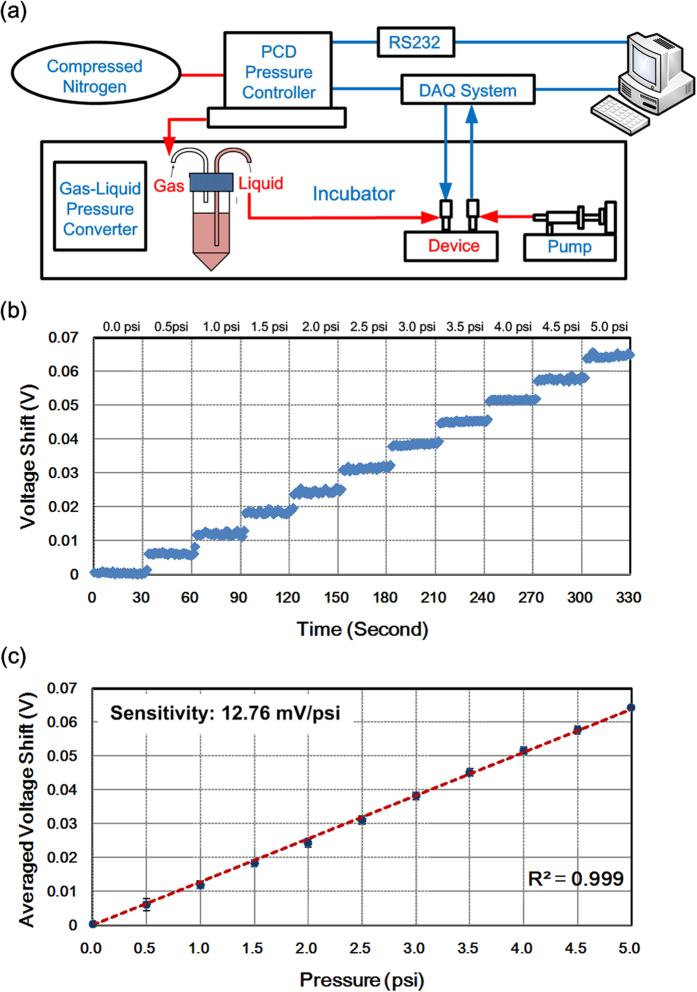
(**a**) Experimental setup for the in-plane elasticity of cell layer measurement. (**b**) Pressure sensor calibration: Raw data of output voltage shifts from the pressure sensor under various applied pressures (0 ~ 5 psi). (**c**) Linear regression of the calibration results. Data are expressed as mean ± sd.

The experimental setup for the in-plane elasticity of cell layers measurement is shown as Fig. 3(a). A data acquisition (DAQ) system (PCIe-6363, National Instruments, Austin, TX) with LabVIEW program (Version 2011, National Instruments) is exploited to apply and record the electrical signals simultaneously. A sinusoidal wave AC voltage signal (±10 V, 2 Hz) is applied to the pressure sensor electrofluidic circuit, and the output voltages across the bridge circuits are detected with a sampling rate of 100 Hz. The root mean square values of output signals with various applied pressures are calculated. The pressure is applied from a nitrogen gas cylinder and regulated with a digital pressure regulator (PCD-15PSIG-D, Alicat Scientific, Inc., Tucson, AZ), which is controlled by the same LabVIEW program. To avoid bubbles in the cell culture channel, the gas pressure is converted to hydrostatic pressure through a gas-liquid pressure adapter before entering the channel. During the experiments, the tubing for pressurization is connected to the inlet of the cell culture channel, and the outlet is injected with growth medium using a computer-controlled syringe pump (Fusion 200, Chemyx Inc., Stafford, TX), with a continuing flow rate of 0.2 μl min^−1^ for nutrition supply. The process for cell culture channel pressurization is achieved by increasing the applied pressure from 0 to 5 psi with increments of 0.5 psi, and each pressure is held for 30 seconds. The pressure is immediately removed, and maintained at 0 psi before the next measurement. The entire cell culture channel pressurization period is about 5 minutes for one measurement to minimize its effect on cell growth. In order to understand the effect of cell adhesion on the change of PDMS membrane’s elasticity and further to estimate in-plane elasticity (Young’s modulus) of cell layers, measurements under three conditions: control (only PDMS membrane), cell measurement (with cells cultured on the membrane), and after cell removal (only PDMS membrane after removing cells) are performed using the same device. The control experiments are conducted first to characterize the sensor performance before cell seeding. The cells are then seeded and cultured to confluence in the device for the cell experiments. After the cell experiments, the cells are removed from the device, and the pressure sensor is characterized to confirm the stable membrane physical properties.

### Cell experiments

In the cell experiments, a human fetal lung fibroblast cell line (MRC-5) is used to investigate its in-plane elasticity, which plays an important role in breathing lungs. MRC-5 cell line is derived from fetal lung tissue, and taken from a 14-week male fetus removed for psychiatric reasons from a 27 year old woman with a genetically normal family history and no sign of neoplastic disease both at abortion and for at least three years afterwards^38^. MRC-5 cells are cultured using a growth medium (MEM medium, Gibco 41090, Invitrogen Co., Carlsbad, CA) with 10% v/v fetal bovine serum (FBS) (Gibco 10082, Invitrogen) and 1% v/v antibiotic–antimycotic (Gibco 15240, Invitrogen), 1% v/v non-essential amino acids (Gibco 11140, Invitrogen), and 1% v/v sodium pyruvate (Gibco 11360, Invitrogen). Stock MRC-5 cells are cultured in a humidified incubator at 37 °C with 5% CO_2_ using the growth medium in T25 cell culture flasks (Nunc 156367, Thermo Scientific Inc., Rochester, NY), and passaged by dissociation with TrypLE™ Express Enzyme (Gibco 12604, Invitrogen).

After the control experiment, the cell culture channel is filled with distilled water and exposed under ultraviolet radiation for 30 minutes for sterilization. Before cell seeding, the cell culture channel is coated with 100 μg ml^−1^ extracellular matrix protein, fibronectin (FC010, EMD Millipore, Billerica, MA), and kept at temperature of 37 °C in the cell incubator overnight. The channels are then washed by the culture medium three times. The MRC-5 cells with a population of 5 × 10^5^ cells in 200 μl of medium are then introduced into the cell culture channel from the inlet and the outlet to ensure channel surface can be over 90% occupied after cell adhesion. The cell measurement is performed 24 hours after cell seeding to assure a confluent MRC-5 cell layer on the PDMS membrane.

To further demonstrate the device capability for the in-plane elasticity measurement over a period for biomedical studies, the cultured MRC-5 fibroblast cells are treated with transforming growth factor-β (TGF-β) for 72 hours to simulate lung fibrosis. TGF-β activation has been implicated in the lung fibrosis, and causes changes in cell phenotypes, including morphological alterations and the expression of α-smooth muscle actin (α-SMA), a marker of myofibroblasts^39^,^40^. In the experiments, the MRC-5 cells cultured inside the device are treated with TGF-β (H8541, Sigma-Aldrich Corp., St. Louis, MO) with concentration of 10 ng ml^−1^ for 3 days. The in-plane elasticity of the transformed MRC-5 cells is then measured using the developed microfluidic device according to the aforementioned procedures. All the in-plane elasticity measurements are performed on the live cells (untreated and TGF-β treated MRC-5 cells). After the cell measurement, the cells are removed from the membrane using the TrypLE™ Express Enzyme (12604-13, Invitrogen). The cell culture channel is washed by the growth medium, and filled with the growth medium for the characterization of the device after cell removal to investigate the device stability during the cell experiments.

In order to confirm the transformation of MRC-5 fibroblasts to myfibroblasts, staining of α-SMA is performed on the MRC-5 cells after the elasticity measurement or on the cells cultured in separate devices. The MRC-5 cells are first fixed by immersing in 4 wt% paraformaldehyde for 30 minutes and Triton X-100 permeation for 20 minutes, followed by blocking in 1 wt% bovine serum albumin (BSA, A2058, Sigma-Aldrich) solution overnight. The MRC-5 cells are than incubated in anti-α-SMA (1:100) antibody (A5228, Sigma-Aldrich) overnight. Afterwards, Alexa Fluor^®^ 488 goat anti-mouse IgG (1:500) antibody (A-11001, Life Technology, Calrsbad, CA) is used to conjugate with the α-SMA antibody.

### Biological AFM measurement

In order to compare the cell elasticity measured using conventional methods, biological AFM (bio-AFM) analysis is performed on the cultured cells. In implementation of bio-AFM, AFM tips are not only widely used for conventional imaging but also suitable for probing mechanical properties of cells^41^,^42^. The tips act as nanoindenters for the acquisition of sample elasticity through AFM measurements. At a certain spot of the cell surface, the normal force applied on a cantilever carrying the tip is consequently recorded as a function of the distance between the tip and the surface, namely, the force-displacement curve. In contact mechanics, the Hertz model considering geometrical effects and local elastic deformation properties of the indented samples has been commonly applied for interrogating the solid elasticity^41^,^43^,^44^,^45^,^46^. In the experiments, elasticity of MRC-5 cells cultured on a conventional 3.5 cm petri-dish (93040, Techno Plastic Products AG, Trasadingen, Switzerland) is characterized using a bio-AFM setup (NanoWizard 3, JPK Instrument AG, Berlin, Germany) for comparison. The collected force-displacement curve corresponding to each pixel of an AFM image is processed to yield the Young’s modulus of the surface membrane of MRC-5 cells for the respective position via optimum fitting with the Hertz model.

## Results and Discussion

### Pressure sensor calibration

To verify the performance of embedded electrofluidic circuit layer, the device is calibrated before the cell experiments. During the calibration, the cell culture channel is pressurized with hydrostatic pressure from 0 psi to 5 psi with increments of 0.5 psi, and each pressure level is maintained for 30 seconds according to the aforementioned procedures. The shift of the output voltage from the pressure sensor is continuously measured by the DAQ system. Figure 3(b) shows a typical time domain output voltage shift under various applied pressures. The voltage shift over time is calculated by subtracting the output voltage across the Wheatstone bridge circuit without applying pressure. In order to estimate the sensitivity of the pressure sensor, the ratio of the output voltage shift to the applied pressure is calculated by linear regression as shown in Fig. 3(c). The result demonstrates excellent sensing linearity (R^2^ > 0.99) of the pressure sensor constructed using the electrofluidic Wheatstone bridge circuit, and the sensitivity is 12.76 mV psi^−1^ for the specific device.

### Cell culture in the device

**Figure 4:**
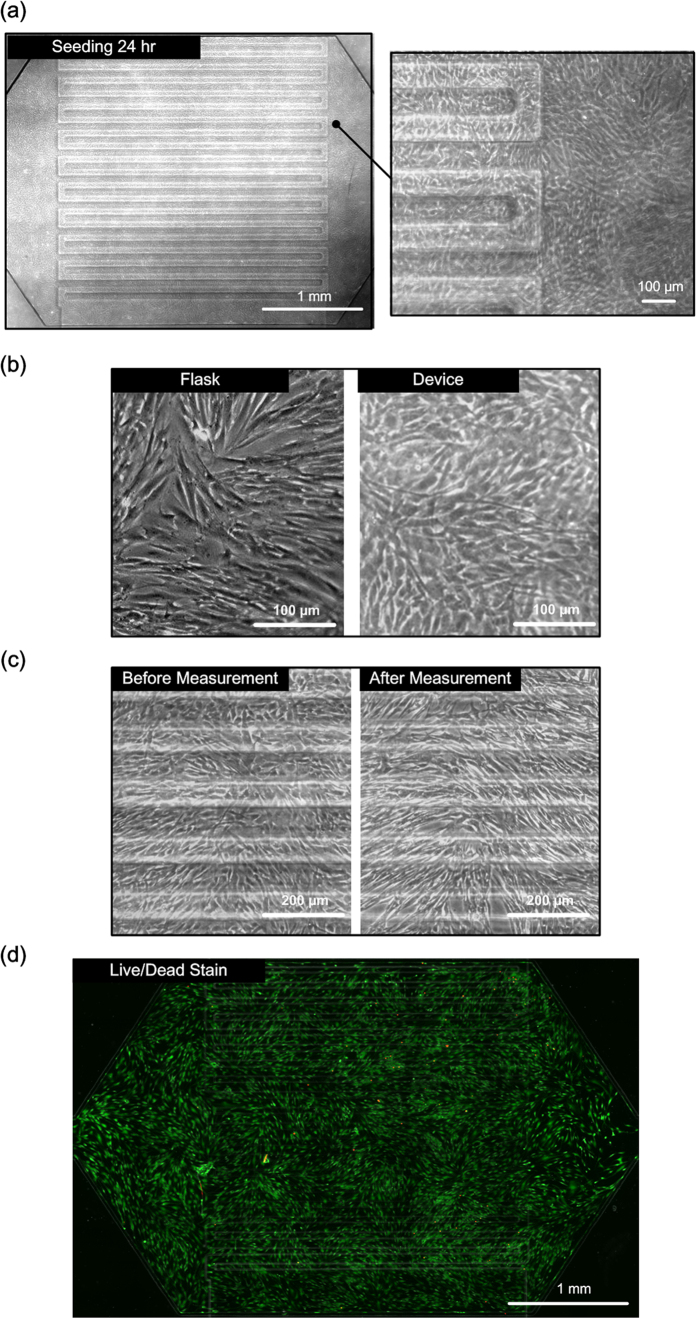
(**a**) Bright field phase images of MRC-5 cells cultured in the microfluidic device for 24 hours. (**b**) Comparison between MRC-5 cells cultured in a T25 flask and in a microfluidic device. (**c**) Cell morphologies before and after the in-plane elasticity measurements. (**d**) Fluorescence image (live/dead stain) of MRC-5 cells cultured in the device after the in-plane elasticity measurement. Live cells show green fluorescence, and dead cells show red fluorescence.

In the cell measurement, the cell culture chamber is seeded with MRC-5 cells. Figure 4 summarizes the experimental results of the cell culture inside the device. Figure 4(a) shows bright field phase images of MRC-5 cells cultured in the cell culture channel 24 hours after seeding. The images demonstrate that the MRC-5 cells attach well onto the PDMS membrane inside the microfluidic device after the ECM protein (fibronectin) surface treatment. Figure 4(b) shows bright field phase images of the MRC-5 cells cultured in a T25 cell culture flask and on the PDMS membrane in the microfluidic device with the ECM protein surface treatment under static conditions. The images show that there is no obvious difference in cell morphologies in the two methods, which suggests the cells remain normal growth inside the device. Furthermore, the bright field phase images of MRC-5 cells shown in Fig. 4(c) before and after the elasticity measurement show no obvious morphological change of cells, and the cells can adhere on the membrane well during the experiments. In order to better investigate the viability of the cells after the experiments, Live/Dead (Calcein AM/Ethidium Homodimer-1) stain from a LIVE/DEAD Viability Cytotoxicity Kit (L3224, Invitrogen) is performed after the cell measurement, and the staining result is shown in Fig. 4(d). In this image, green fluorescence shows live MRC-5 cells, and red fluorescence shows dead cells. The cell viability is estimated to exceed 95%, and the results suggest that the cells experience minimal morphological change and damage during the elasticity measurement experiments.

### Measurement of in-plane elasticity of the cell layers

**Figure 5:**
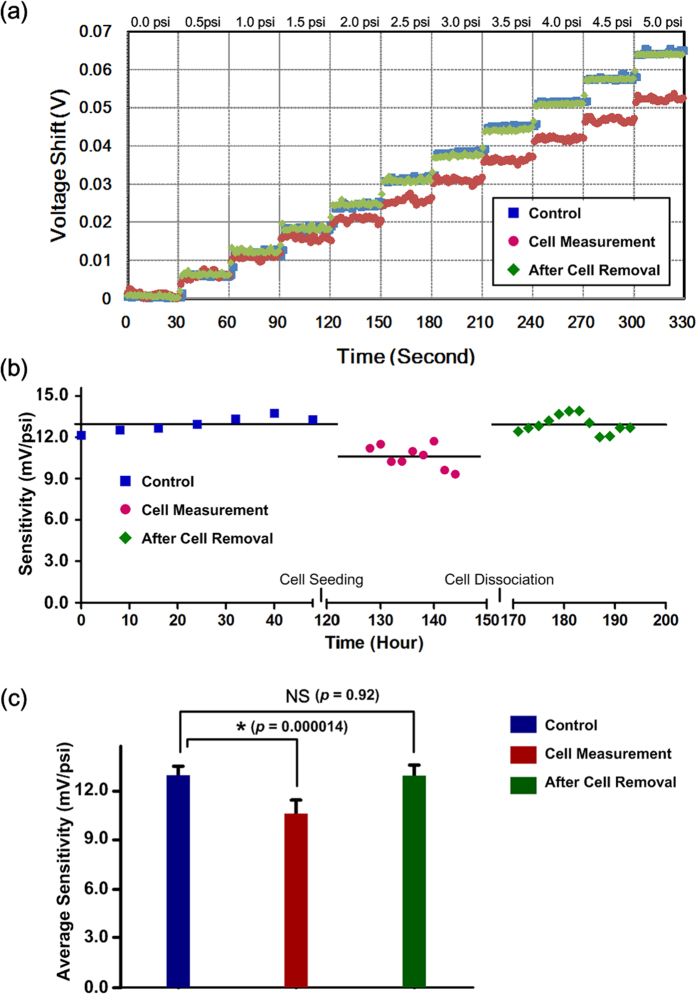
(**a**) Typical raw data of output voltage shifts from the pressure sensor under various applied pressures (0 ~ 5 psi). (**b**) Sensitivity variation during a set of experiment with control, cell measurement, and after cell removal conditions. (**c**) Comparison of the averaged sensitivities under the three experimental conditions. Data are expressed as mean ± sd.

The in-plane elasticity measurement is performed 24 hours after MRC-5 cell seeding into the device to ensure the cells are well attached onto the PDMS membrane surface to form cell layers. Figure 5 shows the electrofluidic pressure sensor measurement results under the three experimental conditions: control, cell measurement, and after cell removal. The raw data of the measured voltage shift under various applied pressure on the cell culture channel according to the aforementioned schemes is plotted in Fig. 5(a). The results show that the measured voltage shift under the same applied pressure is reduced after seeding the cells onto the PDMS membrane due to the increased flexural rigidity of the composite membrane, and the voltage shift is recovered after removing the cells from the membrane. To quantitatively study the pressure sensor output variation, sensitivities calculating the ratios of the measured voltage shift to the applied pressures during the experiments are plotted in Fig. 5(b). From the calculation, the average pressure sensitivity is 12.76 mV psi^−1^ in the control experiments before introducing the cells into the device. The average sensitivity is reduced to 10.46 mV psi^−1^ in the cell measurement experiments, and recovers to 12.73 mV psi^−1^ after cell removal from the cell culture channel. In order to compare the sensitivities from the three different conditions, a statistical analysis, unpaired, two-tailed Student’s t-test is performed, and the results are shown in Fig. 5(c). According to the analysis, the average pressure sensor sensitivity for the cell measurement is statistically lower than that of the control experiment (*p* = 1.42 × 10^−5^), indicating the composite membrane rigidity variation can be successfully observed using the embedded pressure sensor. Furthermore, there is no statistical difference between the average sensitivity of the control and the after cell removal experiments (*p* = 0.92), which suggests the stable PDMS membrane material properties over the entire experiment period.

**Figure 6:**
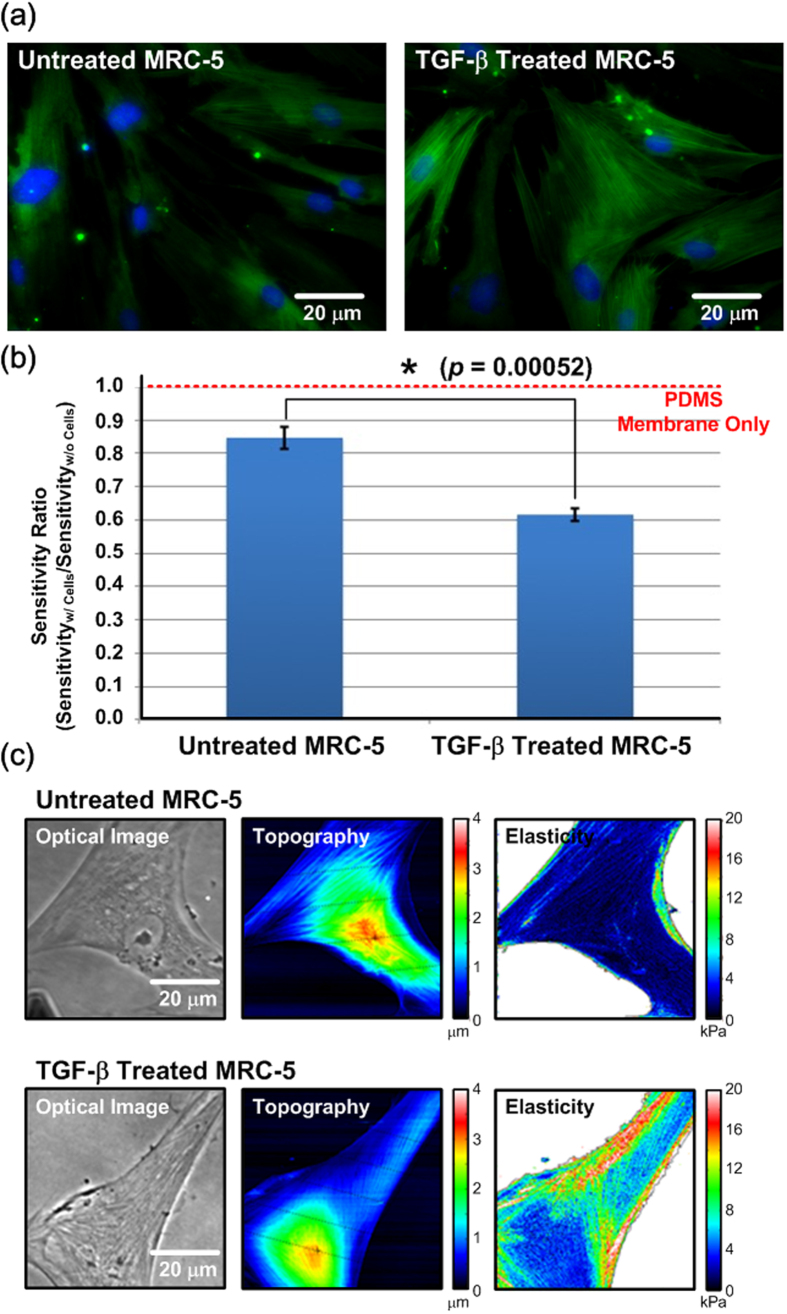
(**a**) Fluorescence images of untreated and TGF-β treated MRC-5 cells (Green: α-SMA; Blue: nuclei). (**b**) Average sensitivity ratios (sensitivity_with cell_/sensitivity_without cell_) of the embedded pressure sensors when culturing untreated and TGF-β treated MRC-5 cells in the microfluidic devices. Data are expressed as mean ± sd (n = 3). (**c**) Topography and elasticity of untreated and TGF-β treated MRC-5 cells measured using AFM.

In the experiments, in-plane elasticity of MRC-5 cells and MRC-5 cells treated with TGF-β to simulate lung fibrosis is measured using the microfluidic device. After a three-day treatment, the MRC-5 cells are fixed and stained with α-SMA to verify the activation of myofibroblasts as shown in Fig. 6(a). The fluorescence images show that the TGF-β treated MRC-5 cells have higher α-SMA expression levels compared to those without the treatment. In order to eliminate the variation between devices for the cell in-plane elasticity measurement, the sensitivity ratio as described in Equation (17) with respect to the control experiment results is calculated for each experiment. Three independent sets of experiments (three devices, and three batches of cells) are conducted in this paper for statistical analysis to confirm the measurement results. Figure 6(b) shows the sensitivity ratios of the devices with untreated and TGF-β treated MRC-5 cells. The results show that the average sensitivity ratio of the untreated MRC-5 cell experiments is approximately 0.85, and the ratio decreases to 0.62 of the treated MRC-5 cell experiments due to the increase of flexural rigidity of the composite membrane. The increase flexural rigidity is resulted from the increase of in-plane elasticity of the MRC-5 cells due to the higher expression level of α-SMA after the TGF-β treatment.

In order to resolve the in-plane elasticity of the cell layers experimentally, Young’s modulus ratio defined as Equation (13) is solved from Equation (17) with the measured sensitivity ratios and cell thickness measured using AFM. In the AFM measurement, the cell morphology and elasticity (Young’s modulus) are characterized using the extended Hertz model for a pyramid probe (PNP-TR, NanoWorld, Neuchâtel, Switzerland) to indent the samples. The AFM characterization results are shown in Fig. 6(c). The average thicknesses of the untreated MRC-5 cells and TGF-β treated MRC-5 cells are estimated to be 1.43 μm and 1.11 μm (n = 3), respectively. There are no statistical differences between the thicknesses of the untreated and treated MRC-5 cells (*p* = 0.39 from unpaired Student’s t-test). The elasticity characterization performed in this paper is highly sensitive to the measured cell layer thickness; however, it is challenging to perform accurate live cell topography measurement over a large area without disturbing cell growth. To further confirm the average thicknesses are representative for the elasticity estimation, AFM morphology characterization is also performed on the untreated and treated MRC-5 cell layers. The results are shown in Fig. S1 in the supplementary information. The thickness histograms suggest that thicknesses of most the cell layer areas are between 0.5 to 1.5 μm, which are similar to their average thicknesses. Consequently, use of the average thicknesses for the elasticity determination should be able to provide reasonably accurate results. According to the thickness characterization, the average Young’s modulus ratio, *n*, can be calculated as 3.45 for the untreated cell layers, and 18.26 for the treated cell layers. The Young’s modulus of PDMS depends on its volume mixing ratio, preparation method, and thickness^47^,^48^,^49^. In order to accurately estimate the elasticity of the exact PDMS membrane within the device, AFM indentation technique is exploited in the experiments. To better characterize the material properties of the PDMS membrane used in the device, measurements of the PDMS membranes exposed in ambient air and the PDMS membranes soaked in the growth medium for overnight are performed for comparison as described in the Supplementary Information. The Young’s modulus of the PDMS soaked in the growth medium, which is determined to be 1.85 ± 0.83 MPa from the AFM experiments, is exploited for the calculation. Therefore, the average in-plane elasticity can be calculated as 6.38 MPa for the untreated MRC-5 cell layers, and 33.78 MPa for the treated MRC-5 cell layers.

For comparison, cell elasticity is also characterized using the AFM, and the average Young’s Modulus are determined to be 4.0 kPa for the untreated MRC-5 cells, and 9.5 kPa for the TGF-β treated MRC-5 cells. Table 1 summarizes the elasticity measured using the developed microfluidic device and conventional AFM method. Comparing to the Young’s modulus characterized on various fibroblast cells in the literatures (e.g. Young’s modulus of chick embryo fibroblasts is 1.2 to 1.7 kPa (tension) and 3 to 4 kPa (compression) by the microplate manipulation, 3T3 fibroblast is 1.7 kPa with adherent junctions, human skin fibroblast is 4.6 to 17 kPa, 3T3 fibroblast is 3 to 12 kPa, and L929 fibroblast is 4 to 5 kPa using an AFM method), the AFM measurement results in this paper are comparable to the reported ones^12^,^14^,^16^,^17^,^21^. However, the in-plane elasticity measured using the microfluidic device is much greater (more than 1,300 times) than the out-of-plane elasticity measured using conventional methods, which suggests the highly anisotropic material properties of cells due to their nature configuration. In the out-of-plane direction, the Young’s modulus measured by the AFM can only represent the elasticity of an area of cell membrane and the cytoplasm enclosed within it. The elasticity is majorly contributed from local microscopic deformation of cell membrane and underlying cytoskeleton. In contrast, in the in-plane direction, cells are connected by various cytoskeletons, which have relative large Young’s modulus. Previous studies have estimated the Young’s modulus of microfilaments, intermediate filaments and microtubules to be 1.8 GPa, 1.2 GPa, and 2 GPa, respectively^50^,^51^,^52^. When cells experience substrate stretching, the tension forces are applied along the cytoskeleton orientation in the in-plane direction. The cytoskeleton can be elongated and the cytoskeleton structure within the entire cell can be macroscopically deformed, which may contribute to the relative large in-plane elasticity. In addition, previous studies mainly discuss cell elasticity of single cells. In contrast, the cell elasticity measurement method developed in this paper involves the elasticity of groups of cells, which is more similar to their physiological arrangements. From the existing studies, elasticity of tissue components ranges from sub-MPa to single GPa, and elasticity of organs and tissue ranges from hundreds of kPa to tens of MPa^53^. Since the cell type characterized in this paper is fibroblast, several proteins may be secreted from the cells for the formation of elastic fibers commonly found in connective tissue. The fibers have been reported to have relatively high elasticity (in order of MPa)^53^,^54^. Therefore, the experimental results demonstrate the in-plane elasticities of cell layers measured using the developed device are reasonable and more close to those of tissues or tissue components.

In the experiments simulating lung fibrosis, both methods show the increase of MRC-5 cell elasticity after myofibroblast transformation due to the higher α-SMA expression level. Interestingly, the increase of the in-plane elasticity of the cell layers is about 429.3%, which is higher than that of the elasticity measured using the AFM (136.2%). The results suggest that the increase of elasticity in both in-plane and out-of-plane direction may not solely depends on the expression level of cytoskeleton protein, α-SMA. The more advanced studies, including: imaging of cytoskeleton arrangement and advanced mechanics model, are required to understand the cell-cell interactions and the effect of α-SMA on elasticity in various directions, and to further investigate the anisotropic physical properties of cells in the future. Since the in-plane elasticity often plays important roles in physiological activities (e.g. vasodilation and lung expansion), the physical effects of the cell transformation (e.g. fibroblast to myfibroblast) can be misled by solely using the AFM characterization. The results suggest the essentialness of the in-plane elasticity characterization of cells, and also demonstrate the developed device may greatly help biologists study fundamental biophysical properties of cells under various physiological conditions.

## Conclusion

The pressure sensor embedded microfluidic device developed in this paper provides an excellent platform that can be exploited to study in-plane elasticity of cells. The pressure sensor can be seamlessly integrated into the device, which makes device fully disposable, and greatly reduces fabrication cost and complexity. Furthermore, the electrical signal output from the sensor enables the fully automated system for long-term measurement. The system can be further scaled up to simultaneously characterize cells under various stimulations for high content testing. According to the pressure calibration results, the Wheatstone bridge constructed using the IL-based electrofluidic circuit provides linear and long-term stable characteristics. In this paper, the pressure sensor is experimentally calibrated and cell compatibility of the device is also confirmed. In order to quantitatively estimate the in-plane elasticity, a theoretical model is constructed in the paper. In the cell experiments, MRC-5 cells without and with TGF-β treatment are cultured and characterized using the developed device, and the results are compared with the AFM measurements. The experimental results confirm the anisotropic physical properties of cells, and demonstrate the importance of the in-plane elasticity measurement. With the demonstrated capabilities, the device can be a powerful platform to study cell physical properties under various physiological conditions, and greatly help biologists investigate essential biophysical properties of cells.

## Additional Information

**How to cite this article**: Lin, C.-H. *et al*. Measurement of in-plane elasticity of live cell layers using a pressure sensor embedded microfluidic device. *Sci. Rep*. **6**, 36425; doi: 10.1038/srep36425 (2016).

**Publisher’s note:** Springer Nature remains neutral with regard to jurisdictional claims in published maps and institutional affiliations.

## Supplementary Material

Supplementary Information

## Figures and Tables

**Table 1:** The comparison of elasticity measured using the developed microfluidic device and the AFM (n = 3 for both methods).

	In-Plane Elasticity by Microfluidic Device	AFM
Untreated MRC-5 (A)	6.38 ± 0.22 MPa	4.00 ± 1.34 kPa
TGF-β Treated MRC-5 (B)	33.78 ± 0.65 MPa	9.46 ± 0.71 kPa
(B)-(A)/(A)	429.3%	136.2%

Data are expresses as mean ± sd.

## References

[b1] Guck J. . Optical Deformability as an Inherent Cell Marker for Testing Malignant Transformation and Metastatic Competence. Biophys. J. 88, 3689–3698 (2005). 15722433 10.1529/biophysj.104.045476 PMC1305515

[b2] Suresh S. . Connections between single-cell biomechanics and human disease states: gastrointestinal cancer and malaria. Acta Biomater. 1, 15–30 (2005). 16701777 10.1016/j.actbio.2004.09.001

[b3] Xu W. . Cell stiffness is a biomarker of the metastatic potential of ovarian cancer cells. PLOS ONE 7, e46609 (2012). 23056368 10.1371/journal.pone.0046609 PMC3464294

[b4] Suresh S. Biomechanics and biophysics of cancer cells. Acta Biomater. 3, 413–438 (2007). 17540628 10.1016/j.actbio.2007.04.002 PMC2917191

[b5] Cross S. E., Jin Y. S., Rao J. & Gimzewski J. K. Nanomechanical analysis of cells from cancer patients. Nat. Nanotechnol. 2, 780–783 (2007). 18654431 10.1038/nnano.2007.388

[b6] Wang G. . Stiffness dependent separation of cells in a microfluidic device. PLOS ONE 8, e75901 (2013). 24146787 10.1371/journal.pone.0075901 PMC3797716

[b7] Evans E. & Yeung A. Apparent viscosity and cortical tension of blood granulocytes determined by micropipet aspiration. Biophys. J. 56, 151–160 (1989). 2752085 10.1016/S0006-3495(89)82660-8 PMC1280460

[b8] Discher D. E., Mohandas N. & Evans E. A. Molecular maps of red cell deformation: hidden elasticity and *in situ* connectivity. Science 266, 1032–1035 (1994). 7973655 10.1126/science.7973655

[b9] Tsai M. A., Frank R. S. & Waugh R. E. Passive mechanical behavior of human neutrophils: effect of cytochalasin B. Biophy. J. 66, 2166–2172 (1994). 10.1016/S0006-3495(94)81012-4 PMC1275942 8075350

[b10] Hénon S., Lenormand G., Richert A. & Gallet F. A new determination of the shear modulus of the human erythrocyte membrane using optical tweezers. Biophys. J. 76, 1145–1151 (1999). 9916046 10.1016/S0006-3495(99)77279-6 PMC1300064

[b11] Guck J., Ananthakrishnan R., Moon T. J., Cunningham C. C. & Käs J. Optical deformability of soft biological dielectrics. Phys. Rev. Lett. 84, 5451 (2000). 10990966 10.1103/PhysRevLett.84.5451

[b12] Thoumine O. & Ott A. Time scale dependent viscoelastic and contractile regimes in fibroblasts probed by microplate manipulation. J. Cell Science 110, 2109–2116 (1997). 9378761 10.1242/jcs.110.17.2109

[b13] Dembo M. & Wang Y.-L. Stresses at the cell-to-substrate interface during locomotion of fibroblasts. Biophys. J. 76, 2307–2316 (1999). 10096925 10.1016/S0006-3495(99)77386-8 PMC1300203

[b14] Ragsdale G. K., Phelps J. & Luby-Phelps K. Viscoelastic response of fibroblasts to tension transmitted through adherens junctions. Biophys. J. 73, 2798–2808 (1997). 9370474 10.1016/S0006-3495(97)78309-7 PMC1181182

[b15] Kuznetsova T. G., Starodubtseva M. N., Yegorenkov N. I., Chizhik S. A. & Zhdanov R. I. Atomic force microscopy probing of cell elasticity. Micron. 38, 824–833 (2007). 17709250 10.1016/j.micron.2007.06.011

[b16] Bushell G. R. . Imaging and force-distance analysis of human fibroblasts *in vitro* by atomic force microscopy. Cytometry 36, 254–264 (1999). 10404976 10.1002/(sici)1097-0320(19990701)36:3<254::aid-cyto16>3.0.co;2-4

[b17] Rotsch C. & Radmacher M. Drug-induced changes of cytoskeletal structure and mechanics in fibroblasts: an atomic force microscopy study. Biophys. J. 78, 520–535 (2000). 10620315 10.1016/S0006-3495(00)76614-8 PMC1300659

[b18] Tomankova K., Kolar P., Malohlava J. & Kolarova H. Mechanical characterization of HeLa cells using atomic force microscopy. Current Microscopy Contributions to Advances in Science and Technology 1, 549–554 (2012).

[b19] Bushell G. R., Cahill C., Myhra S. & Watson G. S. Analysis on human fibroblasts by atomic force microscopy. Methods Mol. Biol. 242, 53–67 (2004). 14578513 10.1385/1-59259-647-9:53

[b20] Starodubtseva M., Chizhik S., Yegorenkov N., Nikitina I. & Drozd E. Study of the mechanical properties of single cells as biocomposites by atomic force microscopy. Microscopy: Science, Technology, Applications and Education 1, 470–477 (2010).

[b21] Wu H. W., Kuhn T. & Moy V. T. Mechanical properties of L929 cells measured by atomic force microscopy: effects of anticytoskeletal drugs and membrane crosslinking. Scanning 20, 389–397 (1998). 9737018 10.1002/sca.1998.4950200504

[b22] Barocas V. H. & Tranquillo R. T. An anisotropic biphasic theory of tissue-equivalent mechanics: the interplay among cell traction, fibrillar network deformation, fibril alignment, and cell contact guidance. J. Biomech. Eng. 119, 137–145 (1997). 9168388 10.1115/1.2796072

[b23] Smith B. A., Clark W. R. & McConnell H. M. Anisotropic molecular motion on cell surfaces. Proc. Natl. Acad. Sci. USA 76, 5641–5644 (1979). 293666 10.1073/pnas.76.11.5641 PMC411705

[b24] Kamotani Y. . Individually programmable cell stretching microwell arrays actuated by a Braille display. Biomaterials 29, 2646–2655 (2008). 18342367 10.1016/j.biomaterials.2008.02.019 PMC2386516

[b25] Moreas C., Chen J.-H., Sun Y. & Simmons C. A. Microfabricated arrays for high-throughput screening of cellular response to cyclic substrate deformation. Lab Chip 10, 227–234 (2010). 20066251 10.1039/b914460a

[b26] Douville N. J. . Combination of fluid and solid mechanical stresses contribute to cell death and detachment in a microfluidic alveolar model. Lab Chip 10, 609–619 (2010). 10.1039/c0lc00251h 21152526

[b27] Huh D. . Reconstituting organ-level lung functions on a chip. Science 328, 1662–1668 (2010). 20576885 10.1126/science.1188302 PMC8335790

[b28] Chang C.-W. . Polydimethylsiloxane-polycarbonate hybrid microfluidic device capable of generating perpendicular chemical and oxygen gradients for cell culture studies. Lab Chip 14, 3762–3772 (2014). 25096368 10.1039/c4lc00732h

[b29] Chang C.-W., Peng C.-C., Liao W.-H. & Tung Y.-C. Polydimethylsiloxane SlipChip for mammalian cell culture applications. Analyst 140, 7355–7365 (2015). 26381390 10.1039/c5an00547g

[b30] Lötters J. C., Olthuis W., Veltink P. H. & Bergveld P. Polydimethylsiloxane as elastic material applied in a capacitive accelerometer. J. Micromech. Microeng. 6, 52–54 (1996).

[b31] Lötters J. C., Olthuis W., Veltink P. H. & Bergveld P. The mechanical properties of the rubber elastic polymer polydimethylsiloxane for sensor applications. J. Micromech. Microeng. 7, 145–147 (1997).

[b32] Hoshino K. & Shimoyama I. An elastic thin-film microlens array with a pneumatic actuator. 2001 MEMS The 14th IEEE International Conference 321–324 (2001).

[b33] Unger M. A., Chou H.-P., Thorsen T., Scherer A. & Quake S. R. Monolithic microfabricated valves and pumps by multilayer soft lithography. Science 288, 113–116 (2000). 10753110 10.1126/science.288.5463.113

[b34] Wu C.-Y., Liao W.-H. & Tung Y.-C. Integrated ionic liquid-based electrofluidic circuits for pressure sensing within polydimethylsiloxane microfluidic systems. Lab Chip 11, 1740–1746 (2011). 21451820 10.1039/c0lc00620c

[b35] Wu C.-Y., Lu J.-C., Liu M.-C. & Tung Y.-C. Integrated electrofluidic circuits: pressure sensing with analog and digital functionalities for microfluidics. Lab Chip 12, 3943–3951 (2012). 22842773 10.1039/c2lc40436b

[b36] Liu M.-C. . Electrofluidic pressure sensor embedded microfluidic device: a study of endothelial cells under hydrostatic pressure and shear stress combinations. Lab Chip 13, 1743–1753 (2013). 23475014 10.1039/c3lc41414k

[b37] Lu J.-C., Liao W.-H. & Tung Y.-C. Magnet-assisted device-level alignment for fabrication of membrane-sandwiched polydimethylisiloxane (PDMS) microfluidic devices J. Microme. Microeng. 22, 75006–75013 (2012).

[b38] Jacobs J. P., Jones C. M. & Baille J. P. Characteristics of a human diploid cell designated MRC-5. Nature 227, 168–170 (1970). 4316953 10.1038/227168a0

[b39] Honda E., Yoshida K. & Munakata H. Transforming growth factor-beta upregulates the expression of integrin and related proteins in MRC-5 human myofibroblasts. Tohoku J. Exp. Med. 220, 319–327 (2010). 20410683 10.1620/tjem.220.319

[b40] Tatlet A. L. & Jenkins G. TGF-β activation and lung fibrosis. Proc. Am. Thorac. Soc. 9, 130–136 (2012). 22802287 10.1513/pats.201201-003AW

[b41] Rico F. . Probing mechanical properties of living cells by atomic force microscopy with blunted pyramidal cantilever tips. Phys. Rev. E Stat. Nonlin. Soft Matter. Phys. 72, 021914 (2005). 16196611 10.1103/PhysRevE.72.021914

[b42] Lu Y.-B. . Viscoelastic properties of individual glial cells and neurons in the CNS. Proc. Natl. Acad. Sci. USA 103, 17759–17764 (2006). 17093050 10.1073/pnas.0606150103 PMC1693820

[b43] Hertz H. Ueber die Berührung fester elastischer Körper. Journal für reine und angewandte Mathematik 92, 156–171 (1881).

[b44] Engler A. J., Sen S., Sweeney H. L. & Discher D. E. Matrix elasticity directs stem cell lineage specification. Cell 126, 677–689 (2006). 16923388 10.1016/j.cell.2006.06.044

[b45] Cross S. E., Jin Y.-S., Rao J. & Gimzewski J. K. Nanomechanical analysis of cells from cancer patients. Nature Nanotechnol. 2, 780–783 (2007). 18654431 10.1038/nnano.2007.388

[b46] Docheva D. . Researching into the cellular shape, volume and elasticity of mesenchymal stem cells, osteoblasts and osteosarcoma cells by atomic force microscopy. J. Cell. Mol. Med. 12, 537–552 (2008). 18419596 10.1111/j.1582-4934.2007.00138.x PMC3822541

[b47] Mills K. L., Zhu X., Takayama S. & Thouless M. D. The mechanical properties of a surface-modified layer on polydimethylsiloxane. J. Mater. Res. 23, 37–48 (2008). 19779588 10.1557/JMR.2008.0029 PMC2749279

[b48] Liu M., Sun J. & Chen Q. Influences of heating temperature on mechanical properties of polydimethylsiloxane. Sens. Actuators, A. 151, 42–45 (2009).

[b49] Liu M., Sun J., Sun Y., Bock C. & Chen Q. Thickness-dependent mechanical properties of polydimethylsiloxane membranes. J. Micromech. Microeng. 19, 035028 (2009).

[b50] Kojima H., Ishijima A. & Yanagida T. Direct measurement of stiffness of single actin filaments with and without tropomyosin by *in vitro* nanomanipulation. Proc. Natl. Acad. Sci. USA 91, 12962–12966 (1994). 7809155 10.1073/pnas.91.26.12962 PMC45560

[b51] Gittes F., Mickey B., Nettleton J. & Howard J. Flexural rigidity of microtubules and actin filaments measured from thermal fluctuations in shape. J. Cell Biol. 120, 923–934 (1993). 8432732 10.1083/jcb.120.4.923 PMC2200075

[b52] Fudge D. S., Gardner K. H., Forsyth V. T., Riekel C. & Gosline J. M. The mechanical properties of hydrated intermediate filaments: insights from hagfish slime threads. Biophys. J. 85, 2015–2027 (2003). 12944314 10.1016/S0006-3495(03)74629-3 PMC1303373

[b53] Akhtar R., Sherratt M. J., Cruickshank J. K. & Derby B. Characterizing the elastic properties of tissues. Mater. Today 14, 96–105 (2011). 10.1016/S1369-7021(11)70059-1 PMC3378034 22723736

[b54] Sherratt M. J. Tissue elasticity and the ageing elastic fibre. Age 31, 305–325 (2009). 19588272 10.1007/s11357-009-9103-6 PMC2813052

