# Supplementary material for: Measurement of in-plane elasticity of live cell layers using a pressure sensor embedded microfluidic device
Source: Sci Rep. 2016 Nov 4;6:36425. doi: 10.1038/srep36425 (PMC5095558; doi:10.1038/srep36425)
Supplement: Supplementary Information [file srep36425-s1.pdf]

Measurement of in-plane elasticity of live cell layers using a pressure sensor embedded microfluidic device

Chien-Han Lin[1,+], Chien-Kai Wang[2,+], Yu-An Chen[3], Chien-Chung Peng[1], Wei-Hao Liao[1], and Yi-Chung Tung[1,*]

[1]Academia Sinica, Research Center for Applied Sciences, Taipei, 11529, Taiwan
[2]Tamkang University, Department of Civil Engineering, New Taipei City, 25137, Taiwan
[3]National Taiwan University, Department of Mechanical Engineering, Taipei, 10617, Taiwan
[*]tungy@gate.sinica.edu.tw
[+]These authors contributed equally to this work

Supplementary Information

S.1 Measurement of Cell Layer Thickness using AFM

To better characterize the thickness distribution of the cell layers, we also perform thickness measurements on the cell layers constructed by untreated and treated MRC-5 cells using the AFM. The measurement results and analyzed thickness histograms are shown in Fig. S1. The histograms show the thickness distribution of the areas within the cells, and suggest large portions of the cell layer thickness are between 0.5 to 1.5 μm for both cell types, which are close to their average thicknesses (1.43 and 1.11 μm for untreated and treated MRC-5 cells, respectively).

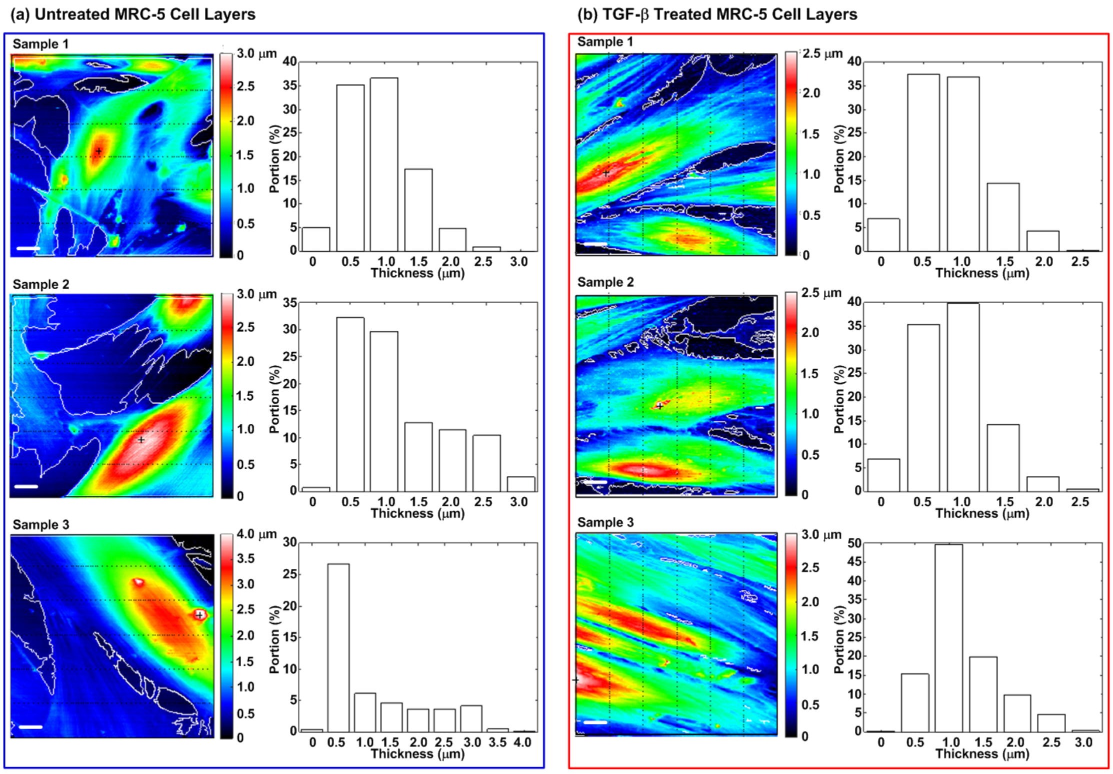

Figure S1. (a) and (b) are the contour plots and histograms of the thicknesses measured on the layers of untreated and TRF-β treated MRC-5 cells, respectively, using AFM. Scale bar is 10 μm.

S.2 Measurement of PDMS membrane Young's Modulus using AFM

The AFM applies the Hertz contact theory for an indenter of four-side pyramid to the indentation force curve to extract the elastic moduli of the PDMS membranes. Through the Hertz model, the relation between the tip force *F* and the tip-sample separation δ of the square-shape indenter is:

$$F = 0.7453 \frac{E}{1-\nu^2} \delta^2 \, tan\alpha \, ,$$

where *E* and ν are the elastic modulus and the Poisson's ratio of the PDMS sample correspondingly, and α is the half face-angle of the pyramid indenter. With both the contact point of the curve and elastic modulus of the sample as floating variables, the measured indentation force versus tip-surface separation data is thus fitted using the least-squares algorithm for determining the desired modulus of the substrate material experimentally. Consequently, using a four-side pyramidal probe with a spring constant of 0.32 N/m and half face-angle of 35 degrees (PNP-TR20, Nanoworld) to indent the substrate, the experimental force curves can be collected in air ambient and the growth medium separately. The measured curves are then fitted with a Poisson's ratio value of 0.5 to further acquire the Young's modulus of the PDMS membranes that are exploited to construct the microfluidic devices developed in this paper. Fig. S2 shows the measurement and analysis results. The results show statistically lower elasticity of the PDMS membrane after soaking in the growth medium, and Young's Modulus are estimated to be 4.06±0.97 and 1.85±0.83 MPa for the PDMS membranes in air ambient and the growth medium, respectively.

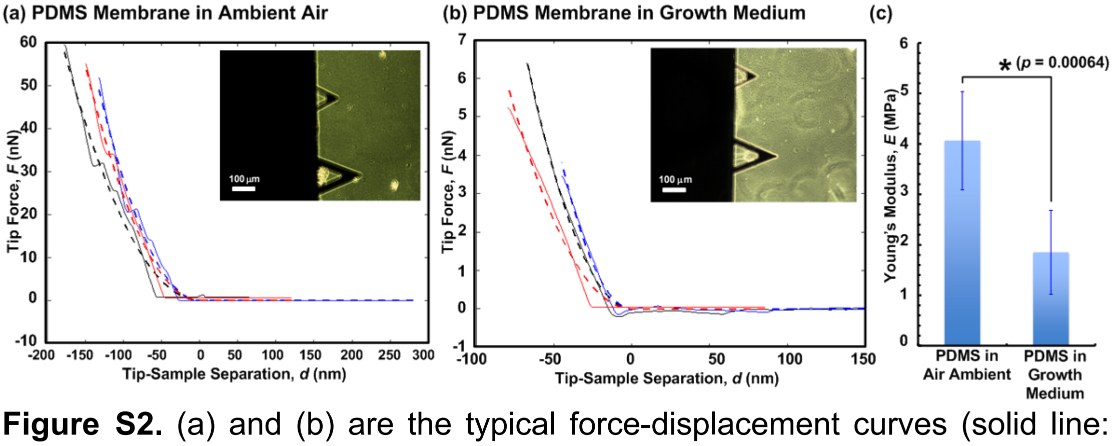

Figure S2. (a) and (b) are the typical force-displacement curves (solid line: raw data; dashed line: Hertz model fitting) obtained from the AFM indentation on the PDMS membranes in air ambient and the growth medium overnight. (c) The calculated Young's modulus from the AFM measurements. Unpaired, Student's t-test is performed to compare the results from the samples (n = 7). Data are expressed as mean ± sd.
